# Nationwide trends and the impact of an oncology hospital network on reducing the burden of thyroid cytology procedures

**DOI:** 10.1002/ijc.35462

**Published:** 2025-05-02

**Authors:** Roos G. F. M. van der Ven, Felice N. van Erning, Daan D. Westra, Ignace H. J. T. de Hingh, Aggie T. G. Paulus, Sanne M. E. Engelen, Bart de Vries, Grard A. P. Nieuwenhuijzen, the OncoZON network

**Affiliations:** ^1^ Department of Research & Development Netherlands Comprehensive Cancer Organisation (IKNL) Utrecht The Netherlands; ^2^ Department of Oncology and Developmental Biology (GROW), Faculty of Health, Medicine and Life Sciences (FHML) Maastricht University Maastricht The Netherlands; ^3^ Department of Health Services Research, Faculty of Health, Care and Public Health Research Institute (CAPHRI), Faculty of Health, Medicine and Life Sciences (FHML) Maastricht University Maastricht The Netherlands; ^4^ Department of Surgery Catharina Hospital Eindhoven The Netherlands; ^5^ School of Health Professions Education (SHE), Faculty of Health, Medicine and Life Sciences (FHML) Maastricht University Maastricht The Netherlands; ^6^ Department of Surgery Maastricht University Medical Center Maastricht The Netherlands; ^7^ Department of Clinical Pathology Zuyderland Medical Center Sittard‐Geleen The Netherlands

**Keywords:** diagnostic procedures, FNA, hospital network, thyroid nodule management, trends

## Abstract

The diagnostic care pathway of thyroid nodules spans multiple institutions. Collaborative networks are important to deal with such pathways that result from centralization and differentiation of care. Despite the high prevalence of thyroid nodules and the increase in cancer diagnoses, most nodules are benign and attributable to overdiagnosis, leading to an increase in fine‐needle aspirations (FNAs). This study assessed the effectiveness of a multi‐hospital network that implemented a unified thyroid care pathway in reducing the number of FNAs without compromising malignancy detection. In this nationwide population‐based cohort study, Bethesda scores were extracted from all thyroid FNA reports from 2010 to 2021 in the Netherlands using text mining. Trends in the number of FNAs and Bethesda scores were visualized for the network and the rest of the country. Joinpoint analyses with the Davies test determined the statistical significance of observed trend changes. Nationwide, FNAs increased by an average of 5.7% annually from 2010 to 2018. In the network, FNAs started to decrease in 2016–2017, coinciding with the care pathway implementation (*p* < 0.001). In contrast, in the rest of the Netherlands, a decline was observed in 2020, potentially attributable to the COVID‐19 pandemic. In both cases, the reduction mainly involved Bethesda categories 1 and 2, without compromising malignancy detection. High‐volume surgical centers seemed to initiate a decline more rapidly compared to non‐high‐volume surgical centers. This study indicates that a unified care pathway within a multi‐hospital network can reduce the number of FNAs without compromising malignancy detection, which could alleviate the burden on both patients and the healthcare system.

## Graphical abstract

What's new?

Increases in diagnostic procedures involving fine‐needle aspiration (FNA) have fueled a rise in incidental thyroid nodule detection. This increase and the frequent need for repeat evaluation of FNA specimens place significant strain on healthcare systems. Here, the authors evaluated the impact of a unified thyroid care pathway implemented in a multi‐hospital network in the Netherlands in 2016–17 on FNA diagnostic burden. Data show that FNAs increased markedly in the Netherlands after 2010 but decreased in the multi‐hospital network utilizing the unified pathway. The findings indicate that coordination across care facilities can lessen diagnostic burdens without compromising thyroid malignancy detection.

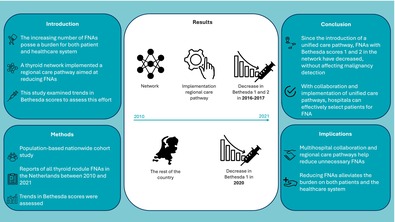

## 1 INTRODUCTION

Thyroid nodules are prevalent, with an estimated occurrence of 60%–65% in the general population.^1^, ^2^ While thyroid cancer has the ninth highest cancer incidence worldwide,^3^ the vast majority of thyroid nodules are small and benign.^4^ Nonetheless, the incidence of thyroid cancer is increasing, with an almost threefold increase in the United States and an incidence of 15.4 per 100,000 per year.^5^ This increasing incidence is largely due to overdiagnosis of asymptomatic low‐risk thyroid nodules detected by increased ultrasonography and PET scan use, rather than a genuine increase in the prevalence of aggressive thyroid cancer.^3^, ^6^, ^7^, ^8^, ^9^, ^10^ Cases found by coincidence, often termed incidentalomas, are supported by autopsy studies, which reveal that many individuals with thyroid cancer were unaware of their condition, never experienced symptoms, and thyroid cancer was not the cause of their death.^3^, ^6^, ^7^, ^8^, ^9^, ^10^

Ultrasound and ultrasound‐guided fine‐needle aspiration (FNA) cytology serve as the cornerstone for thyroid nodule evaluation to assess cancer risk according to the Bethesda Classification.^11^, ^12^, ^13^, ^14^ The overall increase in diagnostic procedures has led to an increase in FNAs of incidentally discovered thyroid nodules.^15^, ^16^ Additionally, approximately 25%–30% of FNA specimens yield indeterminate findings, necessitating repeated evaluations.^17^ This rise in FNAs places a significant burden on the healthcare system, straining both financial and human resources, with estimated diagnostic costs of €1145 per patient.^18^ Moreover, it may cause undue stress and potential harm to patients.^19^, ^20^ Thus, reducing the number of FNAs without compromising malignancy detection is a globally recognized challenge in thyroid cancer management.^21^, ^22^, ^23^ While several international risk stratification systems, such as the radiological TI‐RADS classification, exist to guide FNA decisions,^21^, ^22^, ^23^ a comprehensive implementation of these systems, along with other initiatives, is crucial to alleviating pressures on the healthcare system.

Collaboration in networks emerges as a possible solution to address complex challenges within healthcare systems that surpass the capacity of individual organizations.^24^ In these networks, multiple autonomous organizations, like hospitals, collaborate towards a common goal.^25^ Although many hospitals have been participating in collaborative networks for some time, aimed at sharing medical resources, facilitating knowledge transfer, and exchanging information,^26^, ^27^, ^28^ research shows that networks require a great deal of resources, ~70% of all networks fail, and networks rarely have a significant impact on health outcomes.^29^, ^30^, ^31^, ^32^ More specifically, a previous study on oncology networks demonstrated that simply starting a network does not guarantee change.^33^ In the Netherlands, treatment for thyroid malignancy is centralized in treatment centers due to its rare occurrence, but thyroid diagnostics are conducted in all hospitals.^34^ Therefore, hospitals increasingly work together in regional multi‐hospital networks.^24^ This study examines network effectiveness by evaluating whether a multi‐hospital network that implemented a unified care pathway for thyroid cancer successfully reduced the number of thyroid FNAs without a decrease in the detection of malignancies.

## 2 MATERIALS AND METHODS

FNAs are classified using the Bethesda System for Reporting Thyroid Cytopathology, which categorizes the risk for malignancy (Supplementary file 1).^19^, ^35^, ^36^ There is a widespread recognition of the burden of the increasing number of FNAs, particularly in the context of overdiagnosis leading to further invasive procedures.^19^, ^20^ However, it is important to emphasize that the decision to perform an FNA depends on multiple factors, including ultrasound features like the aspect, dimensions, and growth of the nodule; patient preferences; and clinical characteristics. The complexity and variability in cytological findings necessitate careful and sometimes repeated evaluation to ensure patient safety and accurate diagnosis. This study aimed to provide insights into the trends in Bethesda scores over time, assessing whether a network was able to decrease the number of FNAs without compromising malignancies detected. In this study, a downward trend in Bethesda categories 1 and 2, without a decrease in malignancies, is considered an indicator of network effectiveness in implementing a care pathway to reduce FNAs.

### 2.1 Setting and timeline

In 2021, a crude incidence rate of 5.19 per 100,000 was reported in the Netherlands.^37^ Like in other countries, oncology care in the Netherlands is often organized through regional multi‐hospital oncology networks.^38^ This also applies to thyroid cancer, for which surgical, nuclear, and systemic treatment has been centralized in specialized centers due to its rare occurrence. These centers must meet certain standards, including performing at least 20 thyroid surgeries annually, offering advanced neck dissections for locoregional recurrences, and maintaining a dedicated team comprising at least two thyroid surgeons, two endocrinologists specializing in thyroid cancer, two nuclear medicine specialists, a pathologist with expertise in thyroid cancer, a radiologist, a radiation therapist, and an oncologist.^34^

The regional thyroid network under study, located in the southeast of the Netherlands and serving approximately 10%–15% of the Dutch population, includes 10 hospitals. The network's first meeting took place in October 2015. Since April 2016, various efforts have been made to reduce the number of thyroid FNAs without compromising malignancy detection, such as making regional arrangements (e.g., FNAs must be performed by a dedicated radiologist) and repeatedly discussing best practices in preparation for creating a joint regional care pathway. In June 2016, the network set the explicit goal to reduce the number of FNAs. From January 2017, a unified care pathway was implemented in all organizations involved in the thyroid network, and from February to May 2017, the American College of Radiology Thyroid Imaging Reporting and Data System (ACR TI‐RADS) classification was introduced regionally to categorize thyroid nodules based on ultrasound characteristics, aiming to better predict the risk of malignancy and thereby guide the FNA indication and minimize the need for FNA in low‐risk nodules.^23^

### 2.2 Data sources

For this nationwide population‐based cohort study, data from the Nationwide Network and Registry of Histo‐ and Cytopathology in the Netherlands (PALGA) was used, encompassing all 43 pathology laboratories in the Netherlands.^39^ The registry holds excerpts (i.e., summaries) of all original pathology reports from all pathology laboratories in the Netherlands, including the conclusion text of the involved pathologist. Excerpts of all cytological thyroid FNAs performed between 2010 and 2021 in the Netherlands were obtained at the laboratory level. However, some laboratories serve multiple hospitals. To be able to distinguish excerpts from high‐volume surgical centers and non‐high‐volume surgical centers within the network, laboratories within the network were asked to indicate the originating hospital for each FNA. The conclusion texts from the pathologists were provided in free‐text fields. To extract Bethesda scores from those free‐text fields, a combination of string matching and machine learning techniques was used. Initially, PolyFuzz^40^ and RapidFuzz^41^ were used to identify and extract Bethesda scores by employing matching algorithms to find text segments closely resembling predefined keywords and phrases related to Bethesda scores, such as “Bethesda” and “Bethesda cat II,” including phrases with spelling errors. By setting a minimum similarity threshold, only high‐confidence matches were extracted, thereby reducing the likelihood of false positives. A Bethesda score could not be extracted in 30.9% of cases. The number of reports including the Bethesda score increased over time, from 3.5% in 2010 to 95.2% in 2021. For cases in which the Bethesda score was not extracted via machine learning, a predictive model based on Term Frequency‐Inverse Document Frequency (TF‐IDF)^42^ and logistic regression was utilized. TF‐IDF was used to transform the text data into numerical feature vectors, capturing the importance of words in the context of the documents. The logistic regression model was then trained using the Bethesda scores previously extracted by PolyFuzz and RapidFuzz as training labels. This model predicted the Bethesda scores in the remaining documents based on the open text, thereby complementing the initial extraction process and improving overall coverage. The average accuracy of this prediction of the Bethesda score was 82.5%. To do so, the model was trained on 70% of the excerpts, which included a Bethesda score and validated on the remaining 30% of those excerpts. A more detailed description of the extraction method can be found in Supplementary File 2. The described data extraction methods were performed in Python.

### 2.3 Analysis

Descriptive statistics were used to present the absolute as well as relative number of FNAs within each Bethesda score category. Univariate analyses were performed using chi‐square tests. Since the network‐wide care pathway was implemented in January 2017, univariate analyses were stratified for the period before implementation (2010–2016) and the period since implementation (2017–2021). Previous literature suggests that in hospital networks, high‐volume surgical centers are often early adopters,^33^ implementing new initiatives or care pathways earlier than other centers. Therefore, trends in Bethesda scores between high‐volume surgical centers and non‐high‐volume surgical centers are also presented. High‐volume surgical centers are defined as hospitals that perform both the diagnosis and treatment of thyroid cancer, performing 50 or more thyroid surgeries and 25 or more thyroid cancer surgeries per year. In the network under study, there are three high‐volume surgical centers, which include one academic medical center and two teaching hospitals, and seven non‐high‐volume surgical centers, which consist of teaching hospitals and smaller general hospitals. Looking at the year 2021, the high‐volume surgical centers had an average of 4.291 FTE in service, with an average revenue of €677 million. In contrast, the non‐high‐volume surgical centers had an average of 1.549 FTE and €234 million in revenue.

Plots showing the absolute number of Bethesda scores per category (Bethesda 1 to 6) over time were created for both the Netherlands and the network. For Bethesda categories that visually indicated a trend change in their plots, namely, Bethesda 1, 2, and 3, a joinpoint analysis was conducted to determine whether these deviations were statistically significant, using the segmented package (version 1.3) in R.^43^ The joinpoint regression model estimates the locations of joinpoints in time in which the Davies test indicates significant changes in the slope of the trend. A visual inspection of the trendlines was conducted to verify if the automated estimations aligned with the observed trends. The automated estimation identified only one breakpoint for each trendline. To assess model fit, the model was also adjusted to allow for two breakpoints, with model performance evaluated using Akaike's Information Criterion (AIC). This analysis confirmed that the model with a single breakpoint provided the best fit for each trendline, guiding the final selection. To account for multiple comparisons, a Bonferroni correction was performed using the p.adjust function with the Bonferroni method.^44^ All analyses were conducted using R (version 4.2.3). The significance level adopted was <.05.

## 3 RESULTS

### 3.1 Overall trends in Bethesda scores over time

**FIGURE 1:**
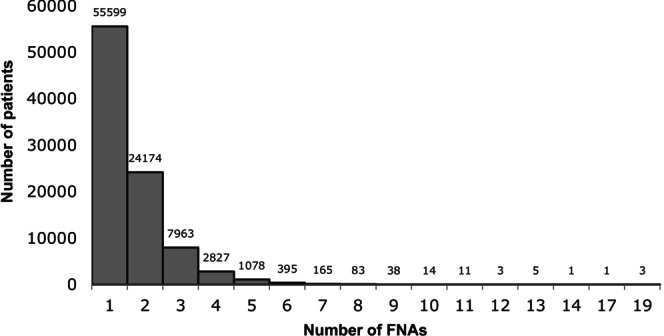
Number of fine needle aspirations per patient.

A total of 149,515 thyroid FNAs were performed in the Netherlands from 2010 to 2021 in 92,360 patients. Nationwide, the median number of FNAs per patient was 1 (IQR 1–2). Most patients (60.2%) underwent only one FNA, while the patient who underwent the most FNAs underwent 19 (Figure 1). In the network, the median number of FNAs per patient was 1 (IQR 1–2) with a maximum of 9 FNAs per patient. Of all 149,515 FNAs performed nationally between 2010 and 2021, 16,165 (10.8%) were performed in the network and 133,350 (89.2%) in the rest of the country. The overall number of FNAs performed in the Netherlands shows a strong upward trend between 2010 and 2018, with an average increase of 640 FNAs per year (5.7%).

**FIGURE 2:**
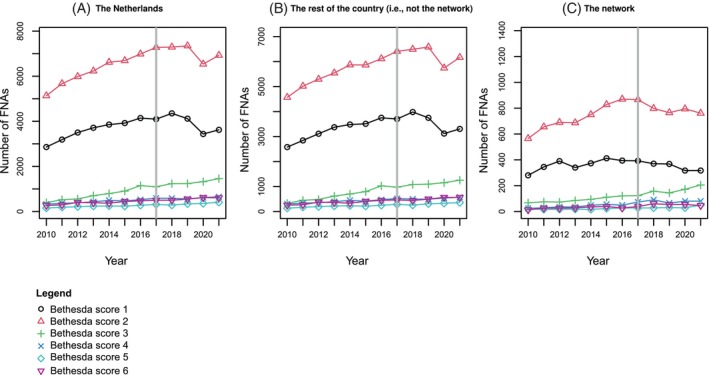
Trends in the absolute number of Bethesda scores over time in (A) the Netherlands, (B) the rest of the country (i.e., not the network), and (C) the network. Black line ●: Bethesda 1. Red line ▲: Bethesda 2. Green line +: Bethesda 3. Dark blue line x: Bethesda 4. Turquoise line ♦: Bethesda 5. Purple line ▼: Bethesda 6. Gray line: time of care pathway implementation.

In total, 30% of FNAs were nondiagnostic or unsatisfactory (Bethesda 1), 52.6% were benign (Bethesda 2), 7.6% appeared to be atypia of undetermined significance or a follicular lesion of undetermined significance (Bethesda 3), 4.0% were diagnosed as (suspicious for) a follicular neoplasm (Bethesda 4), 2.2% were suspicious for malignancy (Bethesda 5), and 3.6% were diagnosed as malignant (Bethesda 6) (Table 1). In 2020, a significant dip in the number of Bethesda 1 and 2 scores is evident, while the number of FNAs with a Bethesda 3 score continues to rise (Figure 2).

**TABLE 1:** Distribution of thyroid FNAs in the Netherlands according to the region of FNA being the network or the rest of the country, stratified for the period of FNA.

	2010–2021						2010–2016						2017–2021					
	The Netherlands		The network		The rest of the country		The Netherlands		The network		The rest of the country		The Netherlands		The network		The rest of the country	
	*n*	%	*n*	%	*n*	%	*n*	%	*n*	%	*n*	%	*n*	%	*n*	%	*n*	%
Bethesda 1	44,806	30.0	4298	26.6	40,508	30.4	25,183	31.2	2534	28.8	22,649	31.5	19,623	28.5	1764	24.0	17,859	29.0
Bethesda 2	78,702	52.6	9030	55.9	69,677	52.3	43,321	53.7	5045	57.3	38,276	53.3	35,386	51.4	3985	54.2	31,401	51.1
Bethesda 3	11,372	7.6	1426	8.8	9946	7.5	5020	6.3	625	7.1	4395	6.1	6352	9.2	801	10.9	5551	9.0
Bethesda 4	5984	4.0	663	4.1	5321	4.0	2982	3.7	275	3.1	2707	2.0	3002	4.4	388	5.3	2614	4.3
Bethesda 5	3238	2.2	302	1.9	2936	2.2	1543	1.9	138	1.6	1405	2.0	1695	2.5	164	2.2	1531	2.5
Bethesda 6	5408	3.6	446	2.8	4962	3.7	2634	3.3	194	2.2	2440	3.4	2774	6.0	252	3.4	2522	4.1
	*p*‐value					<.001	*p*‐value					<.001	*p*‐value					<.001

*Note*: Data are expressed as *n* (%); Bethesda scores according to the Bethesda System for Reporting Thyroid Cytopathology by Cibas & Ali, 2017; *p*‐values based on chi‐square tests.

### 3.2 Trends in Bethesda scores over time in the network (vs. the rest of the country)

**FIGURE 3:**
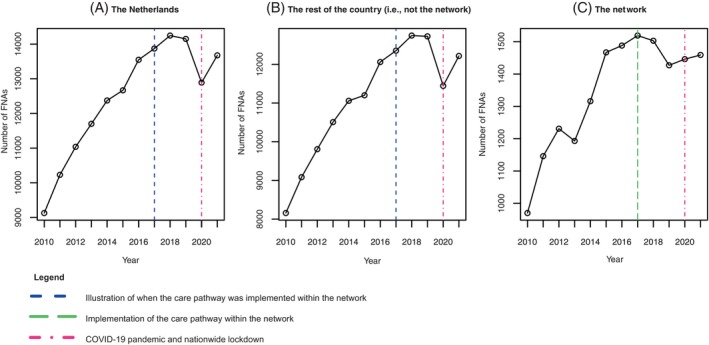
The absolute number of FNAs over time in (A) the Netherlands, (B) the rest of the country (i.e., not the network), and (C) the network. Green line: implementation of the care pathway in this network. Blue line: corresponding timepoint for the rest of the country. Purple line: onset of the COVID‐19 pandemic.

**FIGURE 4:**
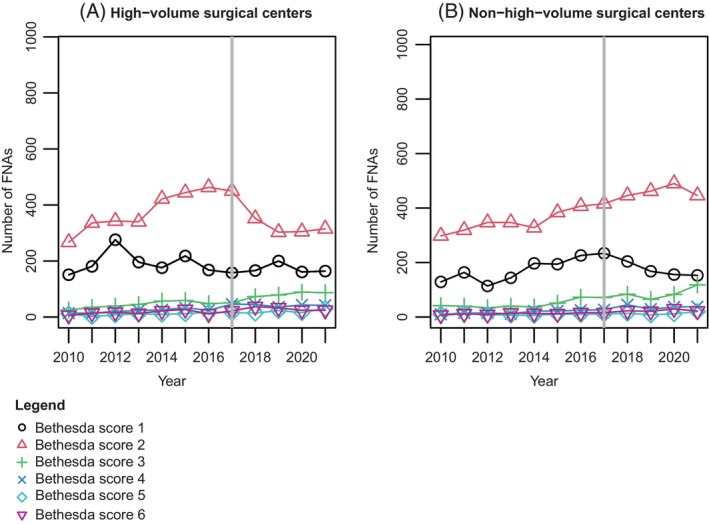
Trends in the absolute number of Bethesda scores over time in (A) high‐volume surgical centers within the network and (B) non‐high‐volume surgical centers within the network. Black line ●: Bethesda 1. Red line ▲: Bethesda 2. Green line +: Bethesda 3. Dark blue line x: Bethesda 4. Turquoise line ♦: Bethesda 5. Purple line ▼: Bethesda 6. Gray line: time of care pathway implementation.

As shown in Figures 2 and 3, both the network and the rest of the country show a clear increasing trend in the overall number of FNAs and in almost all Bethesda score trend lines. However, in the network, a clear deviation in the Bethesda 1 and 2 trend lines appears around 2017, after which a downward trend follows. This is not the case in the rest of the Netherlands, where all trend lines, including the Bethesda 1 and 2 scores, continue to increase over time until 2020. In 2020, the rest of the Netherlands shows a clear and very strong decline, which appears to be barely noticeable in the network. While the trend lines for Bethesda scores 4, 5, and 6 exhibit a stable, slightly upward trend over time, the trend fluctuations are primarily observed in Bethesda scores 1 and 2. In contrast, the trend line for Bethesda score 3 shows a consistent upward movement both within the network and across the rest of the Netherlands. The number of FNAs with a benign conclusion (Bethesda score 2) has been decreasing since 2017 in high‐volume surgical centers but appears to be stable in non‐high‐volume surgical centers. Conversely, a slight decline is observed for non‐diagnostic FNAs in non‐high‐volume surgical centers, which seems to remain stable in high‐volume surgical centers (Figure 4).

### 3.3 Joinpoint regression

As shown in Table 2, in the network, there was an increase of 28.96 FNAs with a Bethesda 1 score from 2010 to 2016. However, this trend was reversed in 2016 (Δ‐49.26, adjusted *p* < .001), leading to a decrease of −20.30 FNAs per year from 2016 to 2020. In the rest of the country, the annual absolute difference was 153.26 from 2010 to 2018, with a decline following the breakpoint in 2018 (Δ‐414.90, adjusted *p* < .001). For Bethesda 2, the network initially showed an increase of 47.09 FNAs per year, but this trend changed to a decline of −21.55 FNAs per year after the breakpoint in 2016 (Δ‐68.65, adjusted *p* < .001). In the rest of the country, there was an annual increase of 243.24 FNAs from 2010 to 2017, which turned into a decline of −182.1 FNAs per year after the breakpoint in 2017 (Δ‐425.34, adjusted *p* = .12). Finally, for Bethesda 3, the network experienced an absolute increase of 9.95 FNAs per year from 2010 to 2019. Following the breakpoint in 2019, there was an annual increase of 33.01 FNAs (Δ23.06, adjusted *p* = .18). The rest of the country did not show significant breakpoints between 2010 and 2021 (adjusted *p* = .36).

**TABLE 2:** Joinpoint regression analysis.

		Segment period	Absolute difference per year, n	Δ of the absolute difference per year	Breakpoint, year (SE)	Davies test‐value	Bonferroni adjusted *p*‐values
Bethesda 1	Network	2010–2016	28.96	‐49.26	2016.44 (0.46)	**<0.001**	**<0.001**
		2016–2020	−20.30				
	Rest of the country	2010–2018	153.26	−414.90	2018.00 (0.42)	**<0.001**	**<0.001**
		2018–2021	−261.64				
Bethesda 2	Network	2010–2016	47.09	−68.65	2016.00 (0.48)	**<0.001**	**<0.001**
		2016–2021	−21.55				
	Rest of the country	2010–2017	243.24	−425.34	2017.62 (0.60)	**0.01**	0.06
		2017–2021	−182.1				
Bethesda 3	Network	2010–2019	9.95	23.06	2019.46 (0.63)	**0.03**	0.18
		2019–2021	33.01				
	Rest of the country	2010–2016	105.43	−47.93	2016.00 (1.07)	0.06	0.36
		2016–2021	57.5				

*Note*: Davies test provides significance of the change in slopes before and after the breakpoint. Bold *p*‐values indicate statistical significance.

Abbreviation: SE, standard error.

## 4 DISCUSSION

This nationwide population‐based study assessed whether a regional multi‐hospital thyroid network was able to reduce the burden of thyroid diagnostics through FNAs by introducing a unified thyroid care pathway. It found that nationwide, the number of FNAs has significantly increased since 2010, particularly in terms of FNAs with a non‐diagnostic or benign outcome (Bethesda scores 1 and 2). However, within the network, a trend reversal was shown since 2016–2017, when the network implemented a unified care pathway to decrease the number of thyroid FNAs. The decrease was (mainly) noted in the absolute number of FNAs with a non‐diagnostic and benign result (Bethesda scores 1 and 2). This early decreasing trend was not present in the rest of the Netherlands. In 2020, however, an abrupt and sharp decline in non‐diagnostic and benign FNAs was observed in the Netherlands, the year of the COVID‐19 outbreak and subsequent nationwide lockdown, but not within the network.

Several studies have documented national or even global trends in thyroid cancer.^8^, ^45^, ^46^ However, comparing the reduction in Bethesda 1 and 2 scores with similar studies from other countries is challenging due to the limited research on trends in Bethesda scores. Most studies present Bethesda score distributions at a single point in time, rather than examining trends over time.^47^, ^48^ These studies often report a higher percentage of benign FNAs (Bethesda 2) and a lower percentage of nondiagnostic or unsatisfactory results (Bethesda 1). This difference can likely be explained by the fact that these studies collect data at the patient level, rather than at the FNA level as in our study. FNAs that were previously non‐diagnostic or unsatisfactory may have been repeated and later classified as benign, thus falling into the Bethesda 2 category. To our knowledge, only one other study has investigated trends in Bethesda scores over time,^49^ but it did not consider the introduction of a network‐wide care pathway, which is a key aspect of our study. This highlights the need for further research on the impact of network‐wide care pathways on Bethesda score trends to assess whether our results are generalizable to other countries.

Therefore, to the best of our knowledge, this is the first nationwide population‐based study investigating trends in FNAs and Bethesda scores. Our findings align with previous research showing a strong increase in FNAs over the past decades, likely due to overdiagnosis of incidental nodules prompted by advancements in highly sensitive imaging techniques.^50^ This study observed a difference in trends between the network and the rest of the Netherlands. While joinpoint analysis indicates a trend change around 2018 for the rest of the country, visual inspection suggests a more pronounced trend change in 2020. This discrepancy is probably due to the limited data points available post‐trend change, as complete data were only available up to 2021. Given the steep trend decrease, nationwide data coverage, and existing literature showing similar COVID‐19 trends,^51^, ^52^, ^53^, ^54^ the 2020 trend dip seems more plausible. This decrease might be explained by the postponement of diagnostics and treatments to contain COVID‐19.^53^, ^54^ While the national decline in FNAs in 2020 is evident, it is not observed within the network, despite its location in the Netherlands' most heavily affected region. This may be explained by the pre‐existing reduction in FNAs, especially in Bethesda category 2 cases, following the introduction of the care pathway in 2017. If this is the case, the pandemic might have masked the true difference, suggesting that the gap in trends between the network and the rest of the country could have been even greater if the COVID‐19 pandemic had not caused such a significant national decline. Additionally, this study observed a slight increase in Bethesda 3 scores in the network. However, the absolute increase is relatively small (∆23.06), and after correction, it is no longer statistically significant (adjusted *p* = .36). Therefore, we expect limited or no substantial clinical relevance compared to the overall decrease in Bethesda 1 and 2 scores.

This study demonstrates that since the implementation of the care pathway, the network has successfully reduced the number of FNAs with Bethesda 1 and 2 scores. The care pathway includes several evidence‐based aspects, which have been refined through best practices and expert discussions during network meetings. According to the pathway, thyroid stimulating hormone (TSH) levels must initially be measured. If TSH falls below the normal reference range, thyroid scintigraphy is conducted. This distinction based on TSH levels is the first step in reducing FNAs and, despite using a different reference range, aligns with previous research showing that scintigraphy for patients with low TSH levels helps avoid FNAs, as hyperfunctioning nodules are rarely cancerous.^55^ For TSH levels within or above the reference range, thyroid ultrasound is performed, and the findings are classified using the ACR TI‐RADS system. The TI‐RADS score then guides the decision on whether an FNA is warranted.^33^ Furthermore, for incidentalomas, FNA is performed only if the nodule is FDG‐positive on a PET scan or if there are additional clinical concerns as determined by the primary physician. While the entire care pathway—including TSH measurements, TI‐RADS classification, and incidentaloma guidelines—has likely contributed to the reduction in FNAs, previous studies have shown that the TI‐RADS classification alone is particularly effective in reducing the number of FNAs.^3^, ^6^, ^7^, ^8^, ^9^, ^10^, ^56^ The substantial impact of TI‐RADS on risk assessment is therefore expected to be the main factor contributing to the observed decrease in Bethesda 1 and 2 scores in this study. A potential concern of a more restrictive FNA policy is the risk of missing malignancies. However, the primary goal of the care pathway, and specifically the implementation of TI‐RADS, is to reduce unnecessary FNAs while maintaining diagnostic accuracy. In the region under study, we have not observed a decline in malignancy detection, and the trend within the network aligns with national trends in regions where the intervention was not implemented, suggesting that fewer FNAs have not led to missed malignancies. Nonetheless, ongoing evaluation remains essential to monitor potential long‐term consequences and ensure the continued safety and effectiveness of this approach.

The data also highlight a notable disparity between high‐volume surgical centers and non‐high‐volume surgical centers within the network, particularly regarding Bethesda score 2. There are two possible explanations for this gap. First, non‐high‐volume surgical centers might be referring fewer patients with benign nodules to high‐volume surgical centers, as was previously observed in another Dutch network.^57^ However, if these centers are indeed referring fewer patients with benign nodules, a decline in Bethesda score 2 would also be expected at these centers. The second possibility is that high‐volume surgical centers may have acted as early adopters of the TI‐RADS classification or might have introduced onsite cytology during FNA procedures (earlier). This observation is in line with findings from another study on the effectiveness of multi‐hospital networks, which similarly noted differences between high‐volume surgical centers and non‐high‐volume surgical centers, likely due to the former being more inclined to adopt new practices early.^18^, ^58^ Although a network aims to function as a unified entity, its initial effectiveness thus appears to rely on its cohesion and its early adopting centers. While implementing a shared care pathway within a multi‐hospital network appears beneficial, its overall impact could be further enhanced if the network succeeds in engaging and integrating all hospitals.

Overdiagnosis by thyroid FNAs, and consequently overtreatment, are huge and widely acknowledged problems, probably secondary to the increase of medical surveillance, improved detection, and incidental findings due to improved and increased ultrasonography and PET scan use.^3^, ^6^, ^7^, ^8^, ^9^, ^10^, ^56^ The aim to reduce the number of FNAs without compromising malignancy detection is therefore widely endorsed, especially given the impact of FNAs on patients as well as the healthcare system.^59^ Previous studies emphasized the physical as well as psychological burden of FNAs on patients, highlighting undue stress or even potential harm.^18^, ^58^ The importance of this reduction is further underscored by the results of this study, showing that 13.6% of patients undergo three or more FNAs, with some extreme cases reaching up to 19 FNAs per patient. In addition to the burden on the patient, the burden on the healthcare system in terms of financial and human resources has been previously documented.^18^ Reducing the number of FNAs, without compromising malignancy detection, may thus have a significant impact on both patients and the healthcare system.

A major strength of this study is its nationwide, population‐based character, allowing for the extraction of trendlines in Bethesda scores over an extended period. The used machine learning techniques combined with the predictive model enabled nationwide coverage. This hybrid approach leverages the strengths of both heuristic and statistical methods, ensuring a more comprehensive and accurate extraction of Bethesda scores from clinical text. This provides a unique insight into FNA trends that, to our knowledge, have not been previously mapped on such a large scale. This study showed that Bethesda scores were rarely recorded in the early years, with documentation increasing over time—a trend observed in both the network and national data. Our dataset includes data from 2010 to 2021. At the end of 2009, the Bethesda Scoring System was introduced, which explains the initial lack of documentation.^60^ As highlighted in the literature, clinical practices often take several years to adopt and fully implement new initiatives.^61^ In our data, by 2015, more than 80% of FNAs had a Bethesda score documented. The gradual increase in Bethesda score usage reflects a natural progression as institutions integrated the system into routine reporting. One limitation of this study is that we focus on trends over time, which may be influenced by other factors during that period, such as the COVID‐19 pandemic or, for example, changes in national healthcare policies, public awareness, or advancements in imaging technology.^51^, ^52^ It is challenging to adjust for these factors, as they affect everyone and could introduce variability that is difficult to account for in the analysis. However, the impact of these factors on the results is expected to be minimal because trends within the network were compared to those in the rest of the country. National healthcare policies affect the entire country, rather than a specific region. Moreover, while demographic differences between regions may exist, they are expected to remain stable over time. Since trends are compared rather than absolute values, sudden demographic changes are unlikely to introduce bias. As a result of the disruption caused by the COVID‐19 pandemic, it was impossible to determine a significant effect of the intervention using multivariate statistics to differentiate the trendlines between the network and the rest of the country. On the other hand, joinpoint analysis revealed a significant trend change in the network around the time of the care pathway implementation. Moreover, this situation provides unique insight into how specialists handle patient selection for FNA during times of crisis, demonstrating that improved patient selection can be achieved without increasing the risk of missing crucial diagnoses (Bethesda scores 4–6). Ideally, a nationwide investigation would determine which patients' nodules were assessed using the ACR TI‐RADS classification, thereby revealing the absolute effect of this specific intervention. Unfortunately, this was not feasible due to privacy regulations, which represents a limitation of this study.

## 5 CONCLUSION

This study shows that a decrease in FNAs correlates with a reduction in non‐diagnostic and benign cases, without compromising the diagnosis of malignant nodules, and can be successfully achieved by a regional hospital network. This presents an effective strategy for implementing these initiatives regionally, thereby reducing the strain on both patients and the healthcare system.

## AUTHOR CONTRIBUTIONS

**Roos G. F. M. van der Ven:** Conceptualization; data curation; formal analysis; investigation; methodology; project administration; resources; validation; visualization; writing – original draft; writing – review and editing. **Felice N. van Erning:** Conceptualization; methodology; supervision; writing – review and editing. **Daan D. Westra:** Conceptualization; methodology; supervision; writing – review and editing. **Ignace H. J. T. de Hingh:** Conceptualization; methodology; supervision; writing – review and editing. **Aggie T. G. Paulus:** Conceptualization; methodology; supervision; writing – review and editing. **Sanne M. E. Engelen:** Conceptualization; methodology; writing – review and editing. **Bart de Vries:** Conceptualization; writing – review and editing. **Grard A. P. Nieuwenhuijzen:** Conceptualization; methodology; writing – review and editing.

## FUNDING INFORMATION

Roos G. F. M. van der Ven receives partial salary support from the OncoZON consortium for a broader project on network research. The consortium had no involvement in the study design, collection, analysis and interpretation of data, writing of the report, and decision to submit the article for publication. No additional funding was obtained other than the partial salary support for the first author.

## CONFLICT OF INTEREST STATEMENT

No potential conflict of interest was reported by the authors.

## ETHICS STATEMENT

Ethical approval for this study was obtained as part of a project on oncology networks from the independent review board of Maastricht University (FHML‐REC/2022/047). Due to the retrospective observational nature of the study, written informed consent was not required under the Dutch Medical Research Involving Human Subjects Act (WMO), which mandates written informed consent only for research involving interventions in the physical or psychological integrity of participants.

## Supporting information


Data S1.

## Data Availability

The data that support the findings of this study are available from the Nationwide Network and Registry of Histo‐ and Cytopathology in the Netherlands (PALGA). Restrictions apply to the availability of these data, which were used under license for this study. Data are available with the permission of the PALGA. Furthermore, all source code is publicly available on GitHub (https://github.com/RGFMvanderVen/Bethesda). Further information is available from the corresponding author upon request.
