# Supplementary material for: Nationwide trends and the impact of an oncology hospital network on reducing the burden of thyroid cytology procedures
Source: Int J Cancer. 2025 May 2;157(6):1120–9. doi: 10.1002/ijc.35462 (PMC12280853; doi:10.1002/ijc.35462)
Supplement: Supplementary file 1 — Data S1. [file IJC-157-1120-s001.pdf]

Supplementary material

Nationwide Trends and the Impact of an Oncology Hospital Network on Reducing the Burden of Thyroid Cytology Procedures

Roos G.F.M. van der Ven, MSc, Felice N. van Erning, PhD, Daan D. Westra, PhD, Ignace H.J.T. de Hingh, MD, PhD, Aggie T.G. Paulus, PhD, Sanne M.E. Engelen, MD, PhD, Bart de Vries, MD, PhD, Grard A.P. Nieuwenhuijzen, MD, PhD, *on behalf of the OncoZON network*

Table of contents

Supplementary file 1 Bethesda System for Reporting Thyroid Cytopathology

Supplementary file 2 Procedure description for extracting Bethesda scores

Supplementary file 1 Bethesda System for Reporting Thyroid Cytopathology

Table S1. The 2017 Bethesda System for Reporting Thyroid Cytopathology*

(adapted from [36])

Score	Definition
Bethesda 1	Nondiagnostic or unsatisfactory
Bethesda 2	Benign
Bethesda 3	Atypia of undetermined significance or follicular lesion of undetermined significance
Bethesda 4	Follicular neoplasm or suspicious for a follicular neoplasm
Bethesda 5	Suspicious for malignancy
Bethesda 6	Malignant

*For a more detailed description of the Bethesda System for Reporting Thyroid Cytopathology see Cibas & Ali, 2017 [36].

Supplementary file 2 Procedure description for extracting Bethesda scores

The conclusion texts form the pathologists were provided in semi-structured free-text fields. To extract Bethesda scores from those free-text fields, a combination of fuzzy string matching and machine learning techniques were used.

Initially, PolyFuzz and RapidFuzz were used to identify and extract Bethesda scores by employing matching algorithm to find text segments closely resembling predefined keywords and phrases related to Bethesda scores, such as "Bethesda" and "Bethesda cat II", including phrases with spelling errors.
The phrases in the free-text fields were compared with the predefined phrases based on their textual similarity. This similarity is defined by the normalized Levenshtein distance (values between 0 and 1) between phrases. For each record, the phrase in the free-text field with the highest similarity to any one of the predefined phrases was extracted. This procedure maximizes the likelihood of extracting phrases with high similarity scores.
To further reduce the likelihood of false positives, a minimum similarity threshold of .8 was set to extract only high-confidence matches. From these resulting phrases, like "Bethesda klasse 2", the actual score (2) was extracted. As the text fields were semi-structured, we found that the score would always be at the end of the given phrase. The threshold of .8 was chosen through two methods. First, manual exploration of the data allowed for quickly viewing whether the output matched the rather short free-text fields. Second, if the wrong phrases were selected we would get an error when extracting the actual score as no integers could be extracted.
Using this procedure, a Bethesda score could not be extracted in 30.9% of cases. The number of reports including the Bethesda score increased over time, from 3.5% in 2010 to 95.2% in 2021.

For cases in which the Bethesda score was not extracted via fuzzy string matching, a predictive model based on Term Frequency-Inverse Document Frequency (TF-IDF) and logistic regression was utilized. TF-IDF was used to transform the text data into numerical feature vectors, capturing the importance of words in the context of the documents. The logistic regression model was then trained using the Bethesda scores previously extracted by PolyFuzz and RapidFuzz as training labels.

This model predicted the Bethesda scores in the remaining documents based on the open text, thereby complementing the initial extraction process and improving overall coverage.
Using 5-fold cross validation, the performance of the model was evaluated on the data extracted through fuzzy string matching. The results showed a macro F1-score of .89 and a micro F1-score of .92.
To further explore the model's performance across classes, a classification report was created by training the model on 70% of the excerpts which included a Bethesda score and validated on the remaining of those excerpt.

bethesda	precision	recall	f1-score	Support
1	0.94	0.90	0.92	7241
2	0.92	0.96	0.94	13252
3	0.93	0.89	0.91	2238
4	0.75	0.76	0.76	1091
5	0.92	0.89	0.90	552
6	0.96	0.90	0.93	554
Accuracy			0.92	24928
Macro avg	0.90	0.88	0.89	24928
Weighted avg	0.92	0.92	0.92	24928

These results demonstrate the cause of the difference between the macro and micro scores, namely that a Bethesda score of 4 is relatively difficult to predict.

To find the cause of this difference, a confusion matrix was created (see Figure S1). This matrix indicates that that Bethesda score 4 is often confused with Bethesda score 2.

Note that other models, including Random Forest and XGBoost were explored but were found to have similar scores. Random Forest showed macro and micro F1-scores of 0.88 and 0.92 respectively. XGBoost showed macro

and micro F1-scores of 0.89 and 0.93 respectively. A logistic regression was chosen as it is most interpretable of these techniques and has similar performance.

Random Forest

bethesda	precision	recall	f1-score	Support
1	0.94	0.91	0.92	7241
2	0.93	0.96	0.94	13252
3	0.94	0.89	0.91	2238
4	0.77	0.75	0.76	1091
5	0.92	0.90	0.91	552
6	0.94	0.91	0.92	554
Accuracy			0.93	24928
Macro avg	0.91	0.89	0.90	24928
Weighted avg	0.93	0.93	0.93	24928

XGBoost

bethesda	precision	recall	f1-score	Support
1	0.94	0.91	0.93	7241
2	0.93	0.96	0.94	13252
3	0.93	0.90	0.91	2238
4	0.76	0.77	0.77	1091
5	0.91	0.91	0.91	552
6	0.94	0.92	0.93	554
Accuracy			0.93	24928
Macro avg	0.90	0.89	0.90	24928
Weighted avg	0.93	0.93	0.93	24928

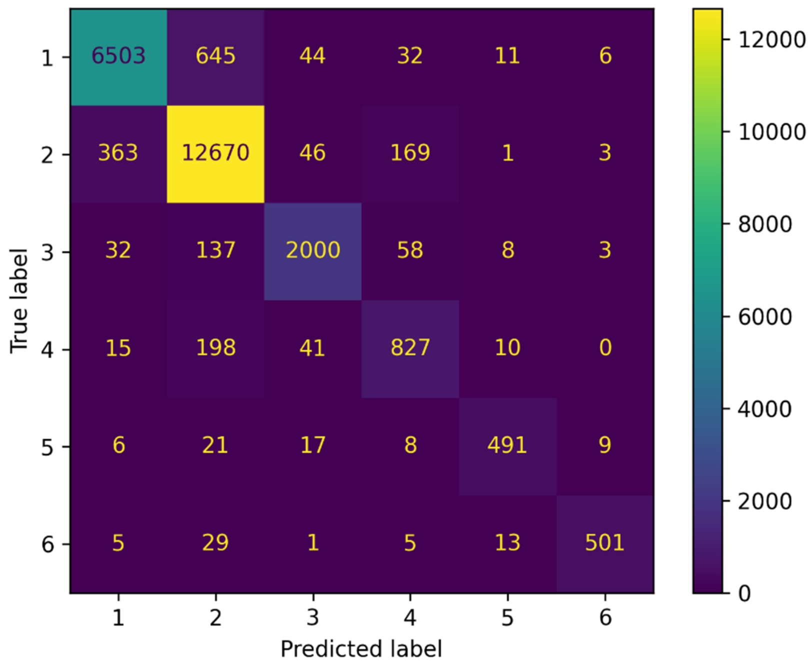

Figure S1 Confusion matrix
